# Separation of Bioactive Compounds in Olive Leaf with a Pyridyl-Functionalized Adsorbent and Hydroalcoholic Solvents

**DOI:** 10.1021/acs.iecr.4c04622

**Published:** 2025-02-26

**Authors:** Elchin Bilalov, Cláudia Martins, Mário Rui P. F. N. Costa, Rolando C. S. Dias

**Affiliations:** † Centro de Investigação de Montanha (CIMO), Instituto Politécnico de Bragança, Campus de Santa Apolónia, 5300-253 Bragança, Portugal; ‡ LSRE, Faculdade de Engenharia da Universidade do Porto, Rua Roberto Frias s/n, 4200-465 Porto, Portugal

## Abstract

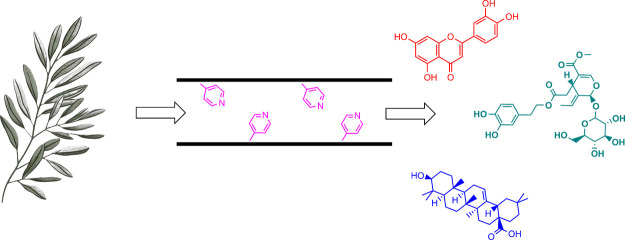

The separation of different types of bioactive compounds in olive leaf is demonstrated with a tailored adsorbent functionalized with pyridyl moieties and sorption–desorption processes developed to use only hydroalcoholic solvents. The competitive binding isotherms of mixtures of vanillic acid, oleuropein, quercetin, maslinic, and oleanolic acids in water/ethanol solvents, with the composition ranging from 50/50 up to 100% ethanol, prove the feasibility of the separation of such different classes of molecules. The bioactive compounds in two industrial olive leaf extracts with different crude compositions were separated with the pyridyl-based polymer particles in packed columns, employing multicycle sorption/desorption processes. A polyphenol-rich extract was subjected to separation, resulting in the isolation of fractions containing varying concentrations of specific compounds. For example, a fraction enriched with oleuropein exhibited a concentration of approximately 80% (an enrichment factor of ∼4 in comparison with the crude extract), while glycosylated flavonoids were present at a concentration of around 60% in another fraction (an enrichment factor of ∼12). Additionally, aglycone flavonoids were present in fractions at a concentration of approximately 83% (an enrichment factor of ∼49). On the other hand, the separation of polyphenols and triterpene acids in an olive leaf extract with a high triterpene content was also demonstrated, with a ratio of flavonoids to triterpenoids of approximately 23 in isolated fractions, as compared to approximately 1 observed in the crude extract. The developed approach yielded luteolin with an enrichment factor of approximately 7. These novel achievements are intended to contribute to sustainability and a circular bioeconomy through the efficient industrial valorization of agricultural byproducts.

## 1 Introduction

A multitude of industries are seeking enhanced solutions with the objective of ensuring the stability and economic viability of biomass valorization across a range of application domains. The energy, food, feed, biotechnological, pharmaceutical, materials, and chemical industries comprise a significant portion of the sectors engaged in this field of study. The valorization of olive leaves represents a meaningful and illustrative case study within this broader category, given the considerable quantity of waste generated by the global olive oil industry. In fact, approximately 4.5 million tons of olive leaves are currently produced worldwide on an annual basis. Despite the significant lignocellulosic content and high concentration of bioactive compounds present in olive leaf biomass, it is currently underutilized. Consequently, recent research has explored the potential of a multivalorization-route biorefinery to valorize olive leaf biomass in a cascade manner.^1^

Concerning bioactive compounds, it is well-known that olive leaves contain different classes of molecules with potential applications in a broad range of industries, namely, polyphenols, such as secoiridoids (e.g., oleuropein), flavonoids (e.g., luteolin, apigenin, quercetin aglycones, and the related glycosides), phenolic acids (e.g., vanillic acid), and phenylethanoids (e.g., tyrosol).^2−4^ Moreover, a high content of triterpenoids (e.g., erythrodiol) and triterpenic acids (e.g., maslinic and oleanolic acids), molecules with proven relevant biological activities, is also observed in the olive leaf.^5,6^

The incorporation of olive leaf bioactive molecules into commercial products, including those in the food, feed, pharmaceuticals, and cosmetic sectors, requires the use of compounds with specified purity or mixtures with tailored compositions. Regardless of the extraction method employed, a mixture of variable complexity is consistently produced, thereby precluding direct utilization of the extracts by the aforementioned industries. This is because, despite the extraction method employed, such as maceration or microwave-assisted extraction with hydroalcoholic or full-organic solvents and supercritical fluids, the resulting mixture is of fluctuating chemical content. In addition to the factors already discussed, the olive variety, geographical origin, and season/year of collection of the olive leaves also introduce variability in the composition of the raw olive leaf extracts. To ensure the stability of the supply to downstream industries, it is necessary to process the olive leaf extracts in order to separate and purify the target compounds.

The most common purification methods for the separation and purification of target compounds in plant extracts include crystallization, distillation, chromatography, and membrane filtration. However, some inherent difficulties are observed when these methods are applied to large-scale processing. The use of crystallization with crude plant extracts is time-consuming and challenging with highly complex mixtures. Furthermore, the need for the selection of optimal solvents and the thermal effects often observed (with possible degradation effects in bioactive compounds) must also be considered. Distillation involves high temperatures, which can also result in conversion and degradation of the target compounds. The use of chromatography requires the employment of costly equipment, with the price per unit of operation increasing exponentially in proportion to the volume to be processed.^7,8^ Membrane filtration is an effective method for separating molecules based on differences in size (such as polysaccharide retention); however, it lacks the ability to selectively separate small molecules in complex mixtures. In contrast, adsorption processes are relatively simple to design, operate, and scale up. Furthermore, sorption/desorption processes are versatile concerning the range of applications, have lower costs, and often present high efficiency.^8^ The aforementioned advantages are stimulating a surge in interest in sorption/desorption processes for the selective separation of a diverse array of compounds, including those present in plant extracts.^8−12^

This work presents a novel approach to the fractionation and purification of polyphenols and triterpenoid acids in olive leaves. The method is based on a sorption/desorption process utilizing a tailored pyridyl-functionalized material acting as an adsorbent. The designed process employs only hydroalcoholic mixtures as solvents, thereby pursuing a more environmentally sustainable and less toxic approach. The adsorbent particles were packed in columns for continuous operation, and knowledge regarding the competitive adsorption of different classes of compounds in olive leaves was acquired through the measurement of multicomponent isotherms. Based on these isotherms, solvent-gradient processes were designed for the fractionation and purification of industrial olive leaf extracts with the pyridyl-functionalized adsorbent. Two different extracts from the industrial processing of olive leaves were considered: a polyphenol-rich extract and a triterpene-rich extract.

## 2 Experimental Section

### 2.1 Materials

Analytical reagent grades for acetonitrile (ACN), acetic acid (AcOH), formic acid, and methanol (MeOH) were bought from Fisher Scientific and those for ethanol (EtOH) from PanReac. Quercetin (hydrate, purity 95%) was supplied by Acros Organics. Oleuropein (pure) was purchased from PanReac and vanillic acid (purity 97%) from Sigma-Aldrich. Oleanolic acid (purity 97%) was bought from Acros Organics, while maslinic acid (purity 92.7%) was provided by NATAC (Alcorcón, Madrid, Spain).

### 2.2 Olive Leaf Extracts

Two different industrial olive leaf extracts provided by NATAC (Alcorcón, Madrid, Spain) were considered for the assessment of their purification with the pyridyl-functionalized particles developed. One extract is rich in polyphenols and contains ∼20% (wt %) of oleuropein (this extract is here named OPA 20), while the other is a side stream of the olive leaf extraction process, containing both triterpenes and polyphenols (this extract is here named VR2 SS1). Additional compositional details on these extracts are provided below, considering the HPLC-DAD analysis.

### 2.3 Pyridyl-Functionalized Adsorbent

The details on the synthesis and characterization of the pyridyl-functionalized adsorbent developed and used in this work have been presented elsewhere.^13^ The 4VP-based particles were packed in a small column with sizes *L* × *D* = 50 mm × 4.6 mm (259 mg), a medium-sized column *L* × *D*=125 mm × 8 mm (1.75 g), and also in a preparative column with dimensions *L* × *D* = 250 mm × 20 mm (25 g). The small column was used with the measurements for the dynamics of adsorption and for the equilibrium isotherms of the mixture containing quercetin, vanillic acid, oleuropein, maslinic acid, and oleanolic acid in the pyridyl-functionalized adsorbent. The medium-sized and preparative columns were used for the purification of olive leaf extracts, as detailed below.

### 2.4 HPLC-DAD Analysis

An HPLC system (KNAUER) consisting of a gradient pump (P6.1 L) equipped with a degasser, an autosampler (6.1 L), a column thermostat (CT2.1), and a DAD (6.1 L) was used in this research. ClarityChrom was the software allowing control of the HPLC system. The chromatographic analysis was performed using an Ascentis C18 (SUPELCO) column with a particle size of 5 μm and dimensions of 25 cm × 4.6 mm. Two different HPLC methods were considered for the quantification of polyphenols and triterpenoids/triterpenic acids in the samples. For polyphenols, a gradient of solvents was used as a mobile phase varying from 100% water–ACN (9:1) to 100% water–ACN (1:9) for 45 min. The mobile phase water pH was adjusted to 3 by using acetic acid. The flow rate of the chromatographic analyses was 1 mL min^–1^, and the temperature of the column was set at 45 °C. For triterpenoids/triterpenic acids, an isocratic analysis was performed using ACN/water (85/15) with 0.05% formic acid as the eluent for 25 min at T = 25 °C.

### 2.5 Dynamics of Adsorption and Equilibrium Isotherms with Standard Molecules

Mixtures of the standard molecules vanillic acid, oleuropein, quercetin, maslinic acid, and oleanolic acid were used to study the competitive adsorption of polyphenols and triterpenic acids in the pyridyl-functionalized particles, namely, the determination of the correspondent equilibrium isotherms with different working conditions (e.g., solvent composition).

Closed-loop adsorption runs were performed with the pyridyl-functionalized particles packed in a small column (*L* × *D* = 50 mm × 4.6 mm, *m*_packed_ = 259 mg) through pumping 25 mL of the mixture of standard compounds at specified concentrations and in selected solvents (EtOH/W mixtures with compositions 50/50, 65/35, 80/20, and 100/0 (v/v)) at a flow rate of 1 mL/min during 3 h. These closed-loop adsorption runs were performed by recycling the column outlet to the feeding flask that was stirred with a magnetic bar. At prescribed time instants, the feeding flask was sampled for determination of the dynamics of adsorption and estimation of the equilibrium approach. The initial solution, intermediate samples, and the final solution were analyzed by HPLC-DAD for quantification of the adsorbed amounts for each compound. After column saturation, the desorption step was performed by percolating 120 mL of MeOH/AcOH (90/10, v/v) through the adsorbent particles, also at a flow rate of 1 mL/min. This desorption step was made considering the open-loop mode (without recycling of column outlet), and the final solution was analyzed by HPLC-DAD for determination of the desorbed amounts for each compound. Comparison between the adsorbed and desorbed amounts was used to verify the mass balance and estimate the uncertainties associated with the measurement of equilibrium isotherms.

Open-loop adsorption runs (without recycling of column outlet) and the correspondent desorption steps were also performed with the polyphenol and triterpene mixtures, following a similar approach to that considered before with polyphenol mixtures.^13^ A good agreement between these two alternative techniques was observed for the measurement of equilibrium isotherms.

### 2.6 Purification of Olive Leaf Extracts in a Preparative Column

The packed preparative column (*L* × *D* = 250 × 20 mm × mm, *m*_packed_ = 25 g) was used for the assessment of the purification of OPA 20 and VR2 SS1 extracts with the pyridyl-functionalized adsorbent. Extracts were dissolved in selected hydroalcoholic solvents (e.g., EtOH/water, 50/50, v/v) at prescribed concentrations (e.g., 3 mg/mL) and loaded onto the column in closed-loop mode at a flow rate of 2 mL/min during a defined total time (e.g., 48 h). During this process, the feeding tank (under magnetic stirring) was sampled for the assessment of the sorption equilibrium (samples were analyzed by HPLC-DAD).

Knauer HPLC pumps (model Azura P 4. 1S, titanium head) with a maximum delivery pressure of 40 MPa and a flow rate in the range of 0.001–10 mL min^–1^ were used to make the flow of feeding solutions and desorption solvents through the particle-packed columns, both for the purification of olive leaf extracts and isotherm determination (previous section). When needed, a column oven was used to define the temperature of the sorption/desorption steps, and the temperature of the feeding solution or desorption solvents was controlled using a thermostatic bath. Enhanced purification of fractions obtained in the preparative column was assessed with the medium-sized column (*L* × *D* = 125 mm × 8 mm, *m*_packed_ = 1.75 g). With this aim, selected fractions were dried and redissolved, at a specified solvent and concentration, for loading in the medium-sized column and subsequent refractionation by desorption with a designed temperature-swing/solvent gradient process.

## 3 Results and Discussion

### 3.1 Pyridyl-Functionalized Adsorbent

The pyridyl-functionalized adsorbent employed in this study was synthesized via the inverse suspension polymerization technique, as previously detailed.^13^ Our approach explores the high binding capacity of pyridyl groups toward numerous polyphenols, even when the impact of hydrophobic effects is minimal, thus enabling the utilization of solvents that permit the processing of olive leaf extracts at high concentrations. Indeed, the special features of pyridine-based polymers are being explored in many branches of science and engineering,^14^ including the valorization of target compounds in agricultural subproducts.^15,16^ In addition to the introduction of pyridyl groups in the polymer backbones, our approach considers the formation of the polymer network with the presence of a template (quercetin) in a molecular imprinting process. Previous studies have demonstrated that molecularly imprinted polymers exhibit an enhanced bonding capacity in comparison to nonimprinted materials, even when the template utilized during the synthesis process was not the intended target molecule. The utilization of a surrogate template during the polymerization process gives rise to alterations in the textural properties of the final materials, accompanied by enhancements in their sorption capacity.^13,15,16^ Related approaches dealing with the development of functional adsorption resins are reported in the literature, such as the introduction of chloromethyl or amino groups to enhance the specific surface area, facilitate hydrogen-bonding interactions, and improve the selectivity of flavone compounds in *Hippophae rhamnoides* L. leaves.^17^

### 3.2 Competitive Sorption and Equilibrium Isotherms for Polyphenols and Triterpenic Acids

Four hydroalcoholic compositions, specifically EtOH/water 50/50, 65/35, 80/20, and 100/0, were considered for the sorption studies. The use of these solvents is “Generally Recognized As Safe (GRAS),” especially in purification processes for nontoxic applications such as food, cosmetics, or pharmaceuticals. Hydroalcoholic solvents have also been employed for the separation of bioactive compounds in olive leaves, as detailed in the following sections. In order to ensure the initial mutual solubility of all standard molecules in the selected solvent, the maximum initial concentration employed was 3 mM when working with 100/0 ethanol, and the minimum initial concentration considered was 0.03125 mM for the 50/50 ethanol/water experiment (see the SI for the concentration ranges used in each experiment). Ethanol, a solvent with minimal mutual solubility limitations, allows for a concentration as high as 10 mg/mL, which is sufficient for the solubilization of an olive leaf extract containing 20% oleuropein (a typical polyphenol-rich industrial extract). This corresponds to a concentration of oleuropein of approximately 3.7 mM. Accordingly, a range for processing a polyphenol-rich olive leaf extract at high concentrations was approached while maintaining comparable solubility for the other compounds. Conversely, the solubility of a triterpene-rich industrial olive leaf extract is approximately 1 mg/mL, which corresponds to a concentration of approximately 0.7 mM in oleanolic acid using an extract with 30% purity as a reference point. Therefore, a typical concentration range for triterpene acids in olive leaf extracts was also approached in the sorption studies.

**Figure 1:**
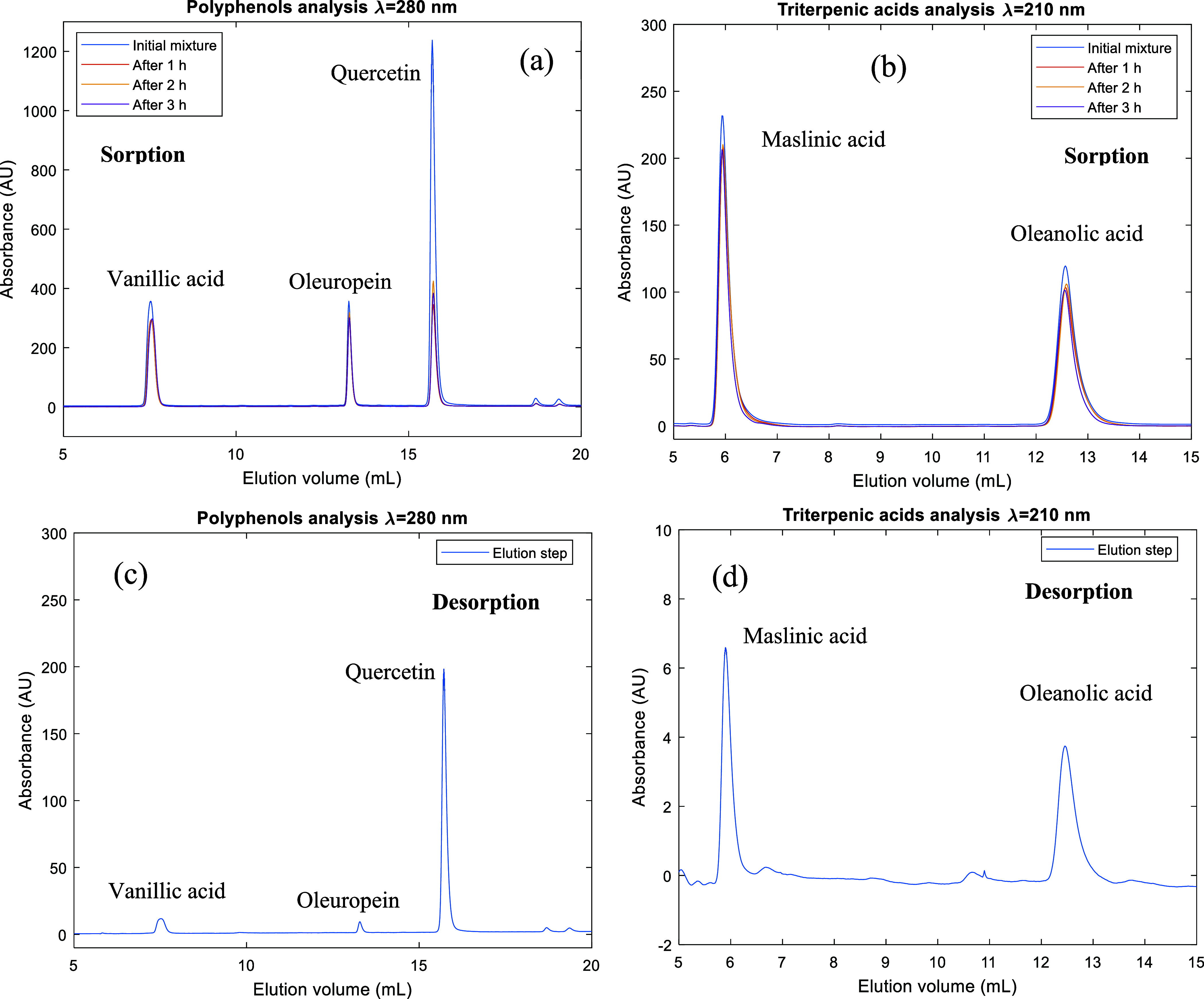
(a,b) HPLC analysis for samples of the liquid phase collected at different time instants during the closed-loop sorption of the mixture of vanillic acid, oleuropein, quercetin, maslinic acid, and oleanolic acid in the pyridyl-functionalized adsorbent particles. (c,d) HPLC analysis for the liquid phase collected after the open-loop desorption with MeOH/AcOH. (a,c) HPLC analysis for polyphenol quantification. (b,d) HPLC analysis for triterpene acid quantification. Experiment at a concentration of 0.5 mM for each compound in EtOH/water 65/35 (v/v).

Figure 1 depicts the methodology employed in this investigation for the examination of competitive adsorption phenomena involving vanillic acid, oleuropein, quercetin, maslinic acid, and oleanolic acid in pyridyl-functionalized adsorbent particles. Samples collected during the column saturation process were subjected to HPLC-DAD for the quantification of the contained compounds. Two distinct analytical methods were employed for the analysis of polyphenols and triterpenes, respectively. Figure 1a,b demonstrates that quercetin is retained to a significantly greater extent than the remaining competitive compounds at equilibrium (3 h running time in the closed loop). Figure 1c,d presents the HPLC analysis for the liquid phase collected after the open-loop desorption of the column with MeOH/AcOH. The desorbed amounts for each compound were obtained from the area of the HPLC peaks, and the results obtained in the sorption cycle were confirmed. As illustrated in Figure 1c,d, the much higher amount of quercetin retained in the pyridyl-functionalized adsorbent particles was confirmed with the desorption step.

**Figure 2:**
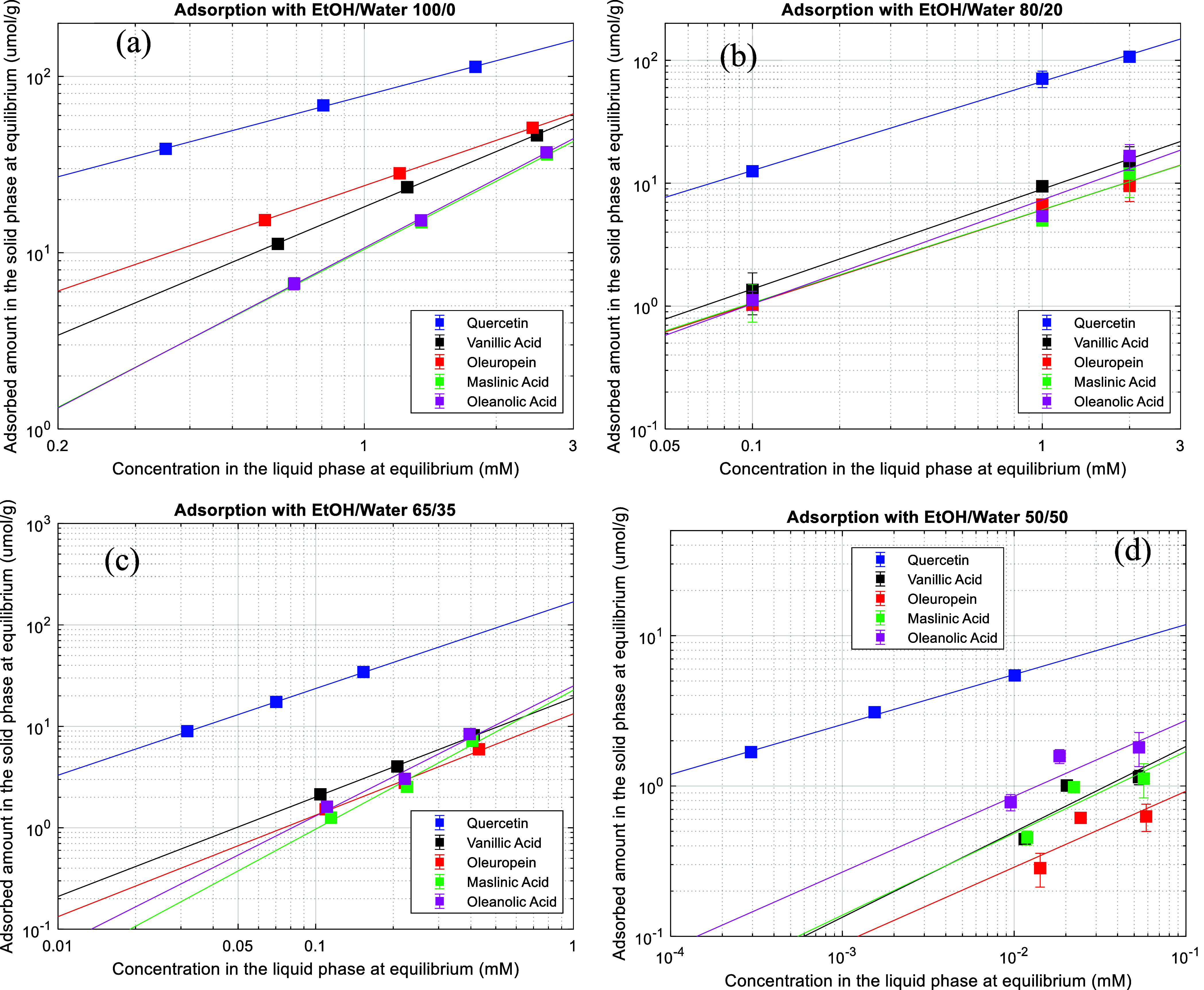
Experimental isotherms measured for the competitive adsorption of vanillic acid, oleuropein, quercetin, maslinic acid, and oleanolic acid in the pyridyl-functionalized adsorbent particles: (a) with EtOH/water 100/0 (v/v) as the solvent, (b) EtOH/water 80/20, (c) EtOH/water 65/35, and (d) EtOH/water 50/50. All the measurements at 25 °C.

The experimental data collected for the different running conditions considered were used to calculate the amount of each compound adsorbed at equilibrium and gain insight into the adsorption isotherms. Figure 2a–d shows these measurements using EtOH/water 100/0, 80/20, 65/35, and 50/50 v/v as loading solvents and *T* = 25 °C. The obtained experimental data show a much higher retention of the polyphenol quercetin compared to vanillic acid and oleuropein polyphenols for all solvent compositions considered. Furthermore, a much higher retention of quercetin compared to the maslinic and oleanolic triterpene acids is also observed. It is also noteworthy to mention the observed effect of hydrophobic interactions on the retention of the different types of compounds, with a significant increase in the retention of oleanolic acid with EtOH/water 50/50 in comparison to maslinic acid, vanillic acid, and oleuropein when using solvent compositions with higher ethanol contents (see Figure 2a–d). It is also noteworthy that the retention of oleuropein, a major compound in olive leaf extracts, decreased when a water-rich solvent was used (see results with EtOH/water 50/50). The hydrophilicity of oleuropein can be exploited for the separation of this bioactive compound from complex mixtures, as will be discussed below. The results presented in Figure 2 suggest the potential for the separation of distinct families of bioactive compounds in olive leaf, including flavonoids, secoiridoids, phenolic acids, and triterpenic acids. The equilibrium adsorption data demonstrate that this separation is feasible when utilizing the developed pyridyl-based adsorbent and employing sorption/desorption processes with hydroalcoholic mixtures. This is applicable not only to the loading stage but also to the solvent-gradient recovery steps.

Considering the objective of designing an effective sorption/desorption process for separation with olive leaf extracts, namely, the definition of the composition of the hydroalcoholic mixtures in the sequence of desorption steps, the experimental information concerning adsorption equilibrium was modeled using a range of isotherm types. Indeed, adsorption isotherm models provide pertinent information regarding the adsorption process, which can be utilized for the design of separation systems that rely on sorption/desorption. In general, isotherms are classified into different groups, namely, (i) adsorption empirical models (e.g., Freundlich isotherm, Redlich–Peterson, etc.), (ii) following Polanyi’s theory, (iii) based on chemical adsorption mechanisms (e.g., Langmuir and Volmer models), (iv) relying on physical adsorption (e.g., BET, Aranovich models), and (v) related to the ion-exchange model.^18^ Also, besides the common individual adsorption models, which show good predictive capabilities with single-component adsorption, many studies consider the development of hybrid and more complex equations, namely, to deal with multicomponent adsorption.^19^ In this work, adsorption models with different degrees of complexity have been considered in order to compare the predictive capabilities for the design of sorption–desorption processes for the separation of bioactive compounds in olive leaf extracts. The individual Langmuir adsorption isotherm, assuming the formation of a monolayer homogeneous surface and no interactions between the adsorbates, was considered as a reference (eq 1, with *j* = 1,2, ···,5 representing the five components considered in the experimental studies). The Freundlich isotherm model (eq 2), considering reversible multilayer adsorption on the heterogeneous surface, was another reference model used here.

Furthermore, the Langmuir model for competitive adsorption (eq 3) and an extended Freundlich isotherm model (eq 4), which also describes competitive adsorption,^19,20^ were considered as potential representations of the present multicomponent adsorption system. The model proposed by Jain and Snoeyink^21^ was here extended to provide a description of the competitive sorption of the five different standard compounds in the pyridyl-functionalized particles. The approach of Jain and Snoeyink^21^ seeks to address some limitations of the extended Langmuir model for competitive adsorption. These include the simplifying assumptions concerning a homogeneous surface for the adsorbent, no interaction between adsorbed species, and equal availability of adsorption sites to all species. Accordingly, additional terms were incorporated into the original Langmuir model for competitive adsorption (eq 3). The objective is to differentiate between the competitive and noncompetitive adsorbed amounts. In the present study, this hypothesis was considered due to the potential for specific imprinted cavities for quercetin in the polymer network, which may influence the competitive and noncompetitive adsorbed amounts.^13^ The following eqs 5 and 6 describe the model used here for the competitive adsorption of quercetin, vanillic acid, oleuropein, maslinic acid, and oleanolic acid in the pyridyl-functionalized particles, with the molecules numbered in the same order (*i* = 1,2,···,5). The first term in eq 5 is employed to quantify the noncompetitive adsorption of quercetin in the adsorbent particles, thereby accounting for any potential molecular imprinting effects. The second term in eq 5 describes the amount of quercetin adsorbed in competition with vanillic acid, oleuropein, maslinic acid, and oleanolic acid. Conversely, eq 6 combines the isotherms for the four remaining compounds, which are assumed to be subject to competition between the five molecules in question.
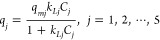
1
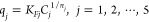
2
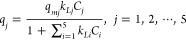
3
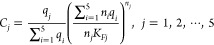
4
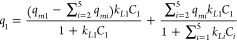
5
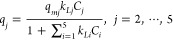
6

Modeling studies of the experimental data with the different isotherms (eqs 1–6) demonstrate good fitting results, both with Langmuir-based and Freundlich-based equations (see fitting parameters in SI Tables S2–S6). However, it should be noted that due to the solubility restrictions mentioned above, the experimental data for the isotherms are situated within a region of low concentration, exhibiting a prevalence of a quasi-linear adsorption regime. Therefore, with the Langmuir models (eqs 1, 3, 5, and 6), especially meaningful are the values for *q*_*mj*_*k*_*Lj*_ that quantify the differences for the binding strength of the different compounds with the different solvents. Regarding the parameters for the Freundlich-based models, the *n* values also indicate the most favorable adsorption of quercetin (*n* > 1 across the entire solvent composition range) in comparison to the other bioactive compounds. Additionally, the observed impact of hydrophobic effects when the water content is increased to 50% (*n* > 1 for all compounds) suggests a higher heterogeneity of the adsorption process due to hydrophobic effects. It is noteworthy that the *n* values are less than 1 for triterpene acids with a high ethanol composition, indicating the potential for separating these compounds from polyphenols in that solvent range.

With regard to the modeling results pertaining to competitive adsorption isotherms (eqs 3–6), the highest degree of discrepancy was observed in the EtOH/water 50/50 solvent composition system. This finding also underscores the challenge associated with these straightforward approaches in accurately capturing the nuances of competitive adsorption under conditions of high heterogeneity, which is influenced by hydrophobic interactions. Moreover, the fitting results with the competitive model, which accounts for specific imprinted cavities regarding quercetin (eqs 5 and 6), indicate that a very small fraction of quercetin is retained without competition in comparison to the competitive model (data in the SI). These results imply that the imprinted cavities are also available for the competitive sorption of the remaining compounds.

**Figure 3:**
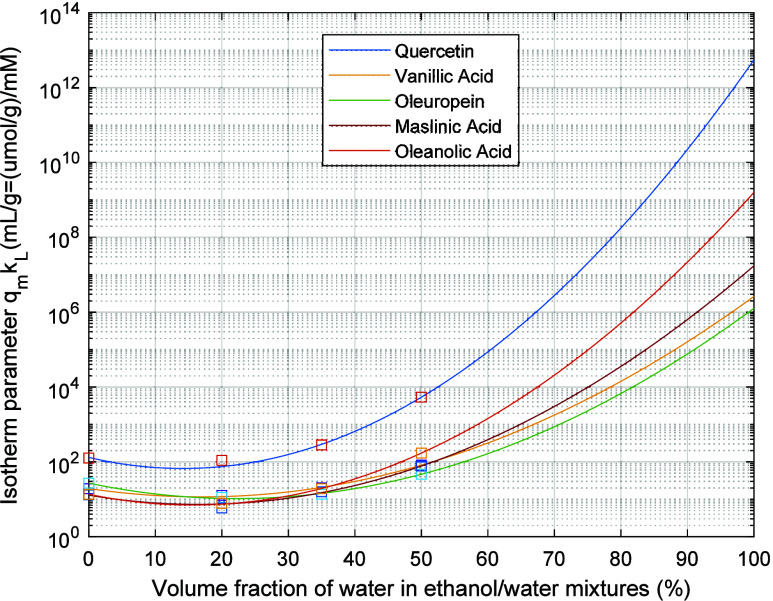
Experimentally estimated values and fitting lines for the change of the parameter *q*_*m*_ × *k*_*L*_ (μmol/g × mM^–1^) with the composition of the solvent considered for the competitive adsorption of vanillic acid, oleuropein, quercetin, maslinic acid, and oleanolic acid in the adsorbent particles. The data for the different compounds were fitted to the model *q*_*m*_ × *k*_*L*_ = 10^(*A*×*W*^2^+*B*×*W*+*C*)^, with *W* representing the volumetric fraction percentage of water in the mixture of ethanol/water.

Subsequently, fitted isotherm models were considered for the design of sorption/desorption processes utilizing the developed pyridyl-based adsorbent for the purification of olive leaf extracts. To this end, the change in model parameters with solvent composition was also fitted, as illustrated in Figure 3 (extrapolation for water contents exceeding 50% was also considered). These fitting lines were afterward employed for the simulation of open-cycle and closed-cycle separation in packed columns, as discussed in the following section.

### 3.3 Simulation of Separation through Successive Solid–Liquid Equilibrium Stages

The experimental data and correspondent fitted isotherm models described above were considered for the simulation of sorption/desorption processes in order to gain knowledge on separation of mixtures with the pyridyl-based particles. This information is afterward used to have simple design rules for processes with real olive leaf extracts, as explored in the next sections. The working with solid–liquid equilibrium stages is illustrated here for the sake of simplicity. These solid–liquid equilibrium stages are experimentally achieved through the closed-loop running configuration, as described above in Section 2.5 (recycling of the packed column outlet to the feeding reservoir containing the liquid phase). For each stage, when the solid–liquid equilibrium is observed, the partition of the different adsorbates between the two phases can be described by the simple mass balance eqs 7 and 8 below. Equation 7 describes a stage starting with clean adsorbent particles and a liquid solution of known composition (adsorption stage), while eq 8 describes the reverse stage, starting with saturated adsorbent particles of known concentration and a blank solvent (desorption stage). In these expressions, *q*_*i*_ represents the amount of compound *i* adsorbed at equilibrium per mass of adsorbent and *C*_*i*_ represents the corresponding concentration in the liquid phase. The mass of adsorbent packed in the column is represented by *m*_ads_, while the volume of the liquid phase is represented by *V*_*L*_. The initial concentration of compound *i* in the liquid phase for an adsorption stage is named *C*_*i*0_, and the initial concentration in the solid particles for a desorption stage is represented by *q*_*i*0_. Assuming the knowledge of the equilibrium isotherms for each component, *q*_*i*_ = *f*_*i*_ (*C*_1_, *C*_2_, ···, *C*_*N*_), as described, for instance, by the models (eqs 1 – 6), the system of algebraic equations below can be solved for the different stages, giving the concentrations in the solid and liquid phases.

Adsorption stage:
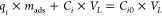
7

Desorption stage:
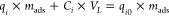
8

**Figure 4:**
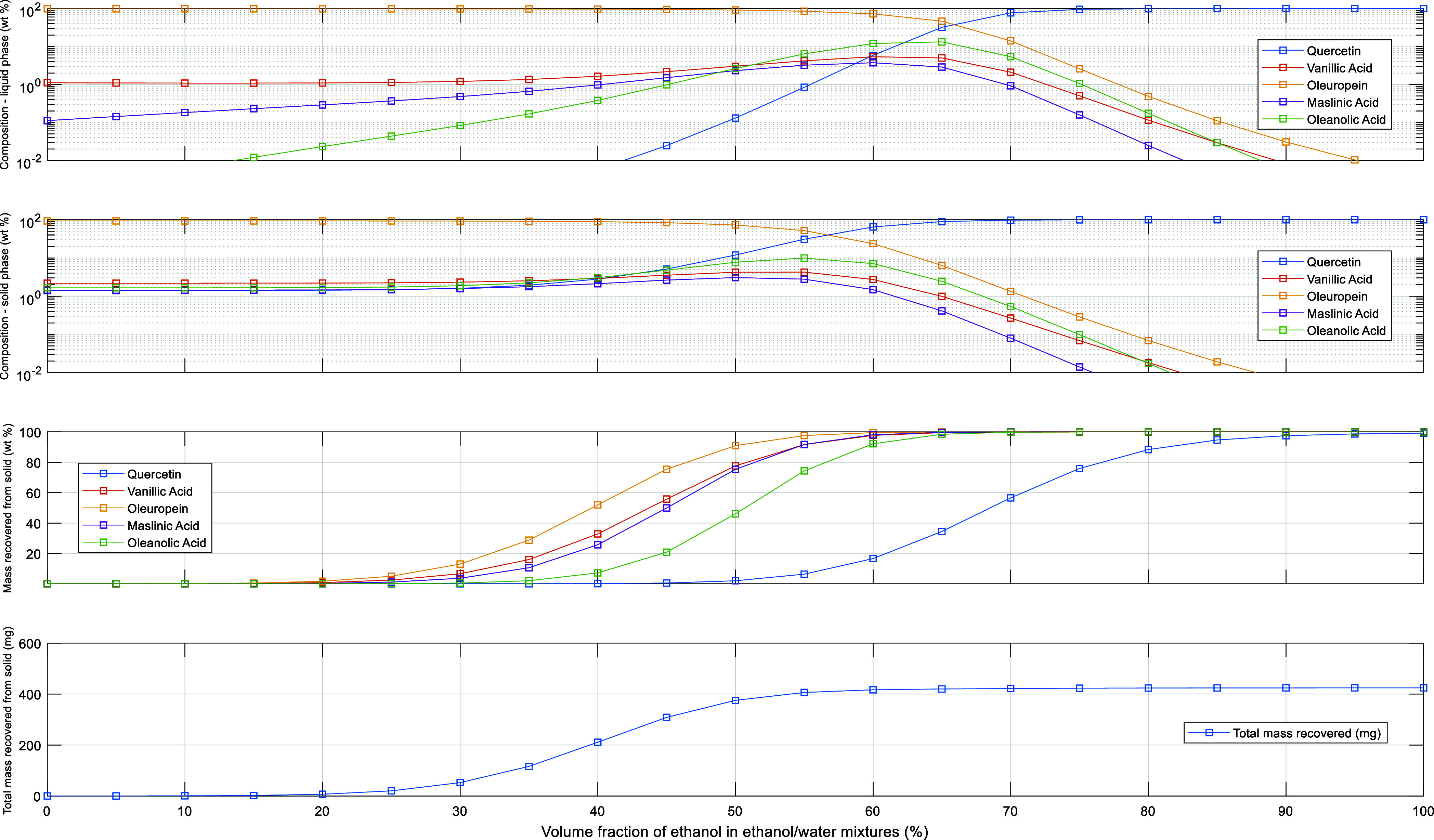
Simulation for a desorption process considering a sequence of solid–liquid equilibrium stages with a change of the ethanol/water solvent composition, starting with pure water and ending with ethanol. In this simulation, the starting of the process is considered for a column packed with 25 g of pyridyl-based adsorbent particles and previously saturated with 600 mL of an extract in ethanol/water 50/50 v/v containing initially oleuropein, vanillic acid, quercetin, maslinic acid, and oleanolic acid at concentrations 1, 0.02, 0.01, 0.013, and 0.013 mg/mL, respectively. These working conditions correspond to the use of an oleuropein-rich olive leaf extract.

Solution of the algebraic eqs 7 and 8 was obtained using the function *fsolve* of MATLAB. Figure 4 presents an example for the simulation of a desorption process considering the change of the ethanol/water solvent composition, starting with pure water and ending with ethanol. A linear adsorption equilibrium with the coefficient described in Figure 3 was assumed in these calculations. Notably, results presented in Figure 4 highlight the separation of oleuropein in the water-rich desorption region, the recovery of vanillic acid, maslinic acid, and oleanolic acid in the range ethanol 40–70%, and the separation of quercetin when working with ethanol >70%. Simulations also demonstrate that the reprocessing of prepurified fractions allows the further enhancement of the separation of the bioactive compounds (e.g., the reprocessing of the fractions collected in the range ethanol 40–70%). Calculations with open-loop desorption follow similar guidelines using the associated partial differential equations solved using the *method of the lines* or the function *pdepe* of MATLAB.^15^

### 3.4 Application to the Separation of Polyphenols and Triterpene Acids in Industrial Olive Leaf Extracts

The potential of the pyridyl-functionalized adsorbent for the separation of different classes of bioactive compounds in olive leaves was demonstrated with the above-described mixture of standard compounds. The knowledge gained was then explored for the fractionation of two industrial olive leaf extracts with a much more complex composition. These extracts were supplied by NATAC (Alcorcón, Madrid, Spain) and have very different compositions in terms of bioactive compounds in the olive leaf. The extract of OPA 20 is a polyphenol-rich mixture containing oleuropein as the main compound (∼20 wt%) and also verbascoside, luteolin glycosides, and many other minority compounds, namely, aglycone flavonoids. The extract VR2 SS1 is very rich in triterpenes, namely, maslinic acid and oleanolic acid, and also contains polyphenols (∼80 wt% of triterpenes in the triterpene and polyphenol mixture).

**Figure 5:**
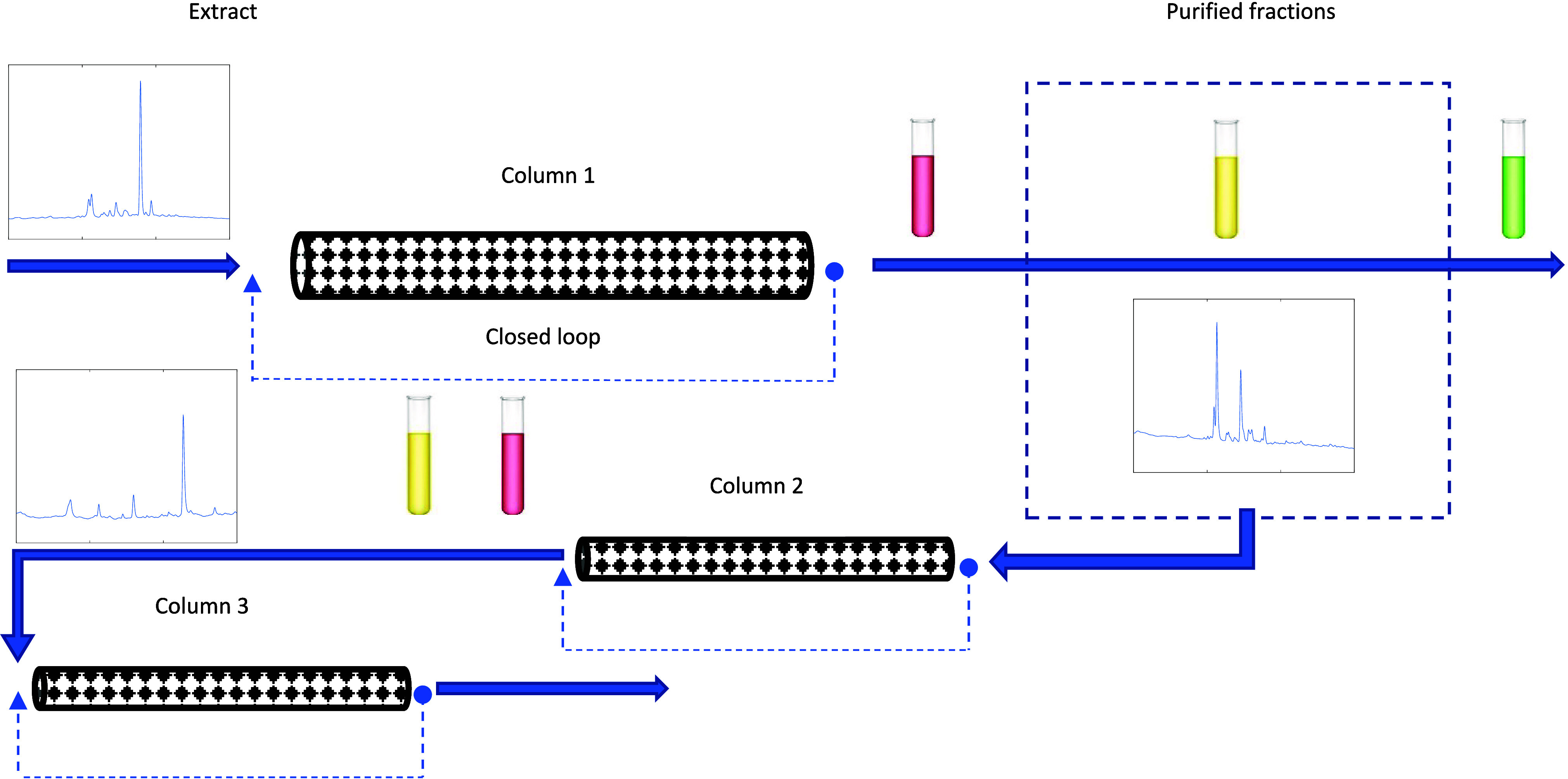
Depiction of the multicycle sorption/desorption process considered in this work for the separation of bioactive compounds in olive leaves with a pyridyl-functionalized adsorbent and using hydroalcoholic solvents. The reprocessing of prepurified fractions was considered for enhancement of separation and purification considering sequential sorption/desorption in packed columns of different sizes and open-loop/closed-loop operation.

The multistep sorption/desorption process developed for the separation of bioactive compounds in industrial olive leaf extracts with a pyridyl-functionalized adsorbent is generically depicted in Figure 5. In summary, the crude extracts are dissolved at a high concentration in a hydroalcoholic solvent (e.g., 5 mg/mL in ethanol/water 50/50 at *T* = 25 °C), and then, the initial sorption step is performed in a first column using open-loop or closed-loop operation (dashed lines in Figure 5). Subsequently, the desorption sequence is conducted with the system equilibrated at a higher temperature (e.g., 45 °C) and a gradient of hydroalcoholic mixtures (e.g., water/ethanol from 100/0 to 0/100). As the desorption sequence progresses, the fractions corresponding to the various solvent compositions are collected separately (see Figure 5). Subsequent separation of the compounds within these fractions can be achieved through their reprocessing by sorption/desorption, as also illustrated in Figure 5. This can be accomplished by utilizing the same column (column 1) or other columns packed with the pyridyl-functionalized adsorbent (e.g., columns 2 and 3 in Figure 5).

**Figure 6:**
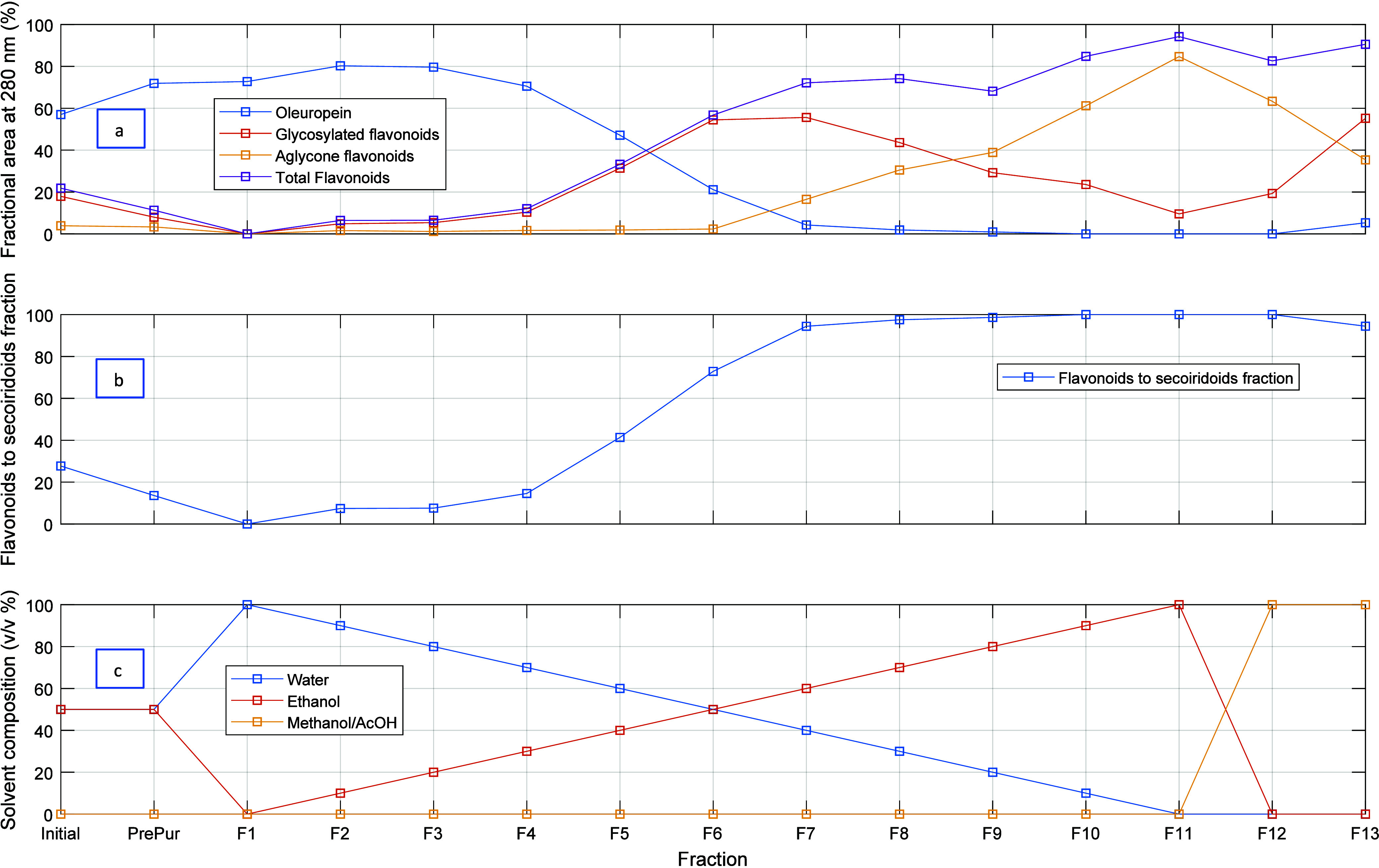
Illustration of a typical desorption step considered in the multicycle sorption/desorption fractionation of the OPA 20 olive leaf extract using a pyridyl-functionalized adsorbent and hydroalcoholic solvents. The process involves a solvent gradient desorption at 45 °C starting from the column previously loaded with the crude extract or a preprocessed fraction (see Figure 5). A total of 13 different fractions were produced in this run. (a) Composition of each fraction, (b) separation between secoiridoids and flavonoids, and (c) solvent gradient used in this experiment.

Figure 6 shows the results of the separation of polyphenols in the OPA 20 olive leaf extract using the multistep sorption/desorption process developed in this research. Thirteen different fractions were obtained in this experiment, whose composition with respect to secoiridoids, namely, oleuropein, glycosylated flavonoids (e.g., luteolin-7-O-glucoside and apigenin-7-O-glucoside), and aglycone flavonoids (e.g., luteolin, quercetin, apigenin) is shown to change with the hydroalcoholic solvent used along the desorption of the pyridyl-functionalized polymer particles. Note the high separation achieved for oleuropein (∼80% fractional area in the HPLC chromatogram at 280 nm, as shown in Figure 6a) in the fractions desorbed with ethanol/water 15/85 and 20/80 (F2 and F3 in Figure 6c). On the other hand, the separation of glycosylated flavonoids reaches a maximum of ∼60% for fractions collected with ethanol/water 50/50 and 60/40, while for high alcoholic fractions (e.g., ethanol 100), the separation of aglycone flavonoids is observed (∼80% in F11). Figure 6b shows an overview of the separation between secoiridoids and flavonoids achieved with the developed process, with the production of highly enriched fractions for secoiridoids in the desorption range with low alcohol content (F1–F3, ethanol 0–20%) and fractions containing mostly flavonoids for the range working at higher alcohol contents (F7–F13).

**Figure 7:**
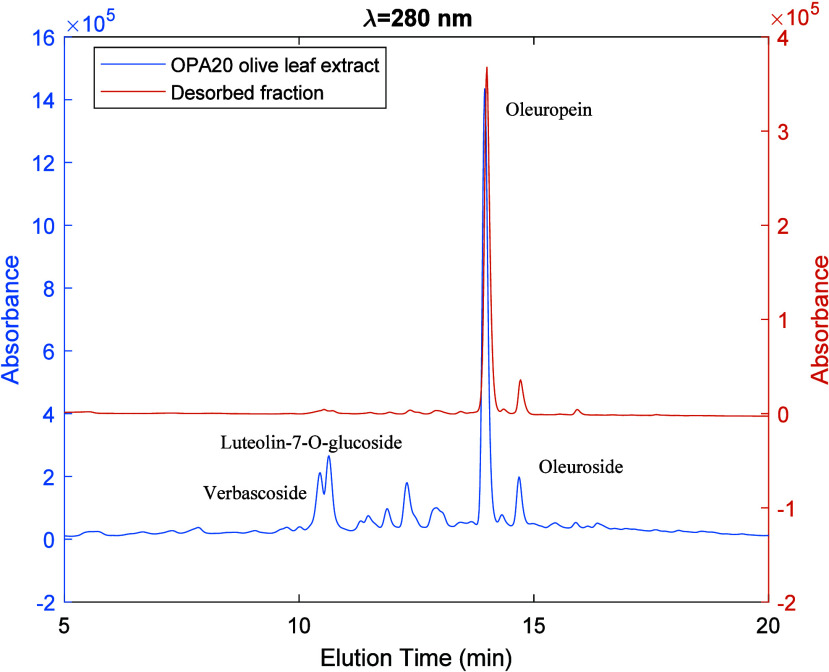
HPLC-DAD chromatograms (λ = 280 nm) for the crude OPA20 olive leaf extract (blue line) and a fraction desorbed with ethanol/water 20/80 v/v (orange line) according to the solvent gradient method illustrated in Figure 6. These results demonstrate the enrichment of oleuropein in the desorbed fraction as compared with the initial leaf extract.

**Figure 8:**
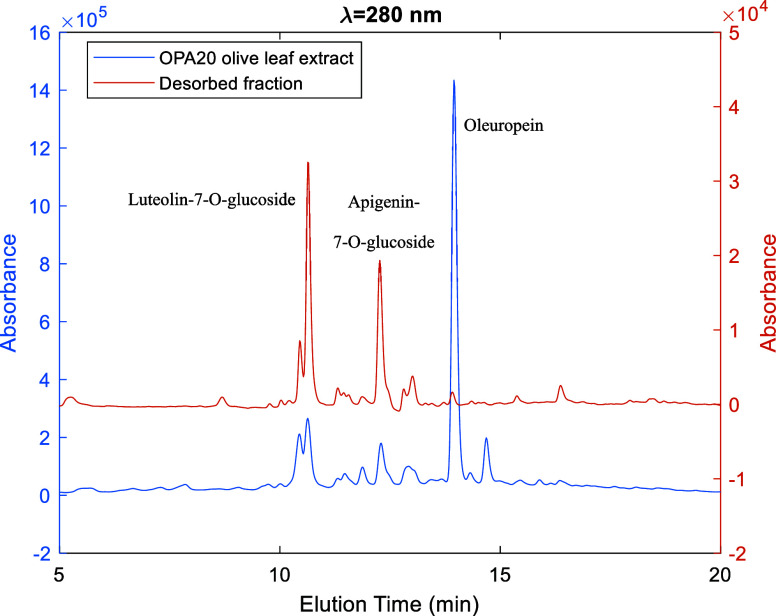
HPLC-DAD chromatograms (λ = 280 nm) for the crude OPA20 olive leaf extract (blue line) and a fraction desorbed with ethanol/water 50/50 v/v (orange line) according to the solvent gradient method illustrated in Figure 6. These results demonstrate the enrichment of glycosylated flavonoids (luteolin-7-O-glucoside, apigenin-7-O-glucoside) in the desorbed fraction compared to the initial leaf extract.

**Figure 9:**
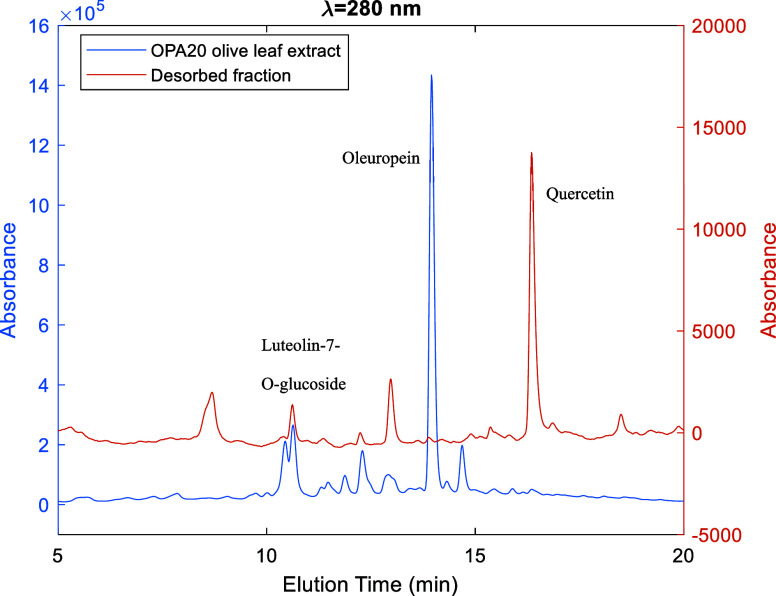
HPLC-DAD chromatograms (λ = 280 nm) for the crude OPA20 olive leaf extract (blue line) and a fraction desorbed with ethanol (orange line) according to the solvent gradient method illustrated in Figure 6. These results demonstrate the enrichment of aglycone flavonoids (quercetin) in the desorbed fraction compared to the initial leaf extract.

Figures 7–9 illustrate the HPLC-DAD analysis of selected desorbed fractions, thereby demonstrating the results presented in Figure 6. Figure 7 presents a comparison of the HPLC-DAD chromatograms (λ = 280 nm) for the crude OPA20 olive leaf extract and the fraction desorbed with ethanol/water 20/80 v/v according to the solvent gradient method illustrated in Figure 6. These results demonstrate that the desorbed fraction has been significantly enriched in oleuropein compared with the initial leaf extract. Indeed, the fractional area for oleuropein in the separated fraction is approximately 80%, whereas in the original extract, it is approximately 20%. Figure 8 presents a similar comparison for the crude OPA20 olive leaf extract and the fraction desorbed with ethanol/water (50/50 v/v), which displays the enrichment of glycosylated flavonoids. The desorbed fraction exhibited a significantly higher concentration of luteolin-7-O-glucoside and apigenin-7-O-glucoside (representing 60% for glycosylated flavonoids, in comparison to approximately 5% in the crude olive leaf extract). Furthermore, Figure 9 illustrates the significant enrichment of aglycone flavonoids, specifically quercetin, in a fraction desorbed with ethanol. It is notable that the measured concentration of aglycone flavonoids in the separated fraction was approximately 83%, in comparison to the concentration of approximately 1.7% observed in the olive leaf extract.

**Figure 10:**
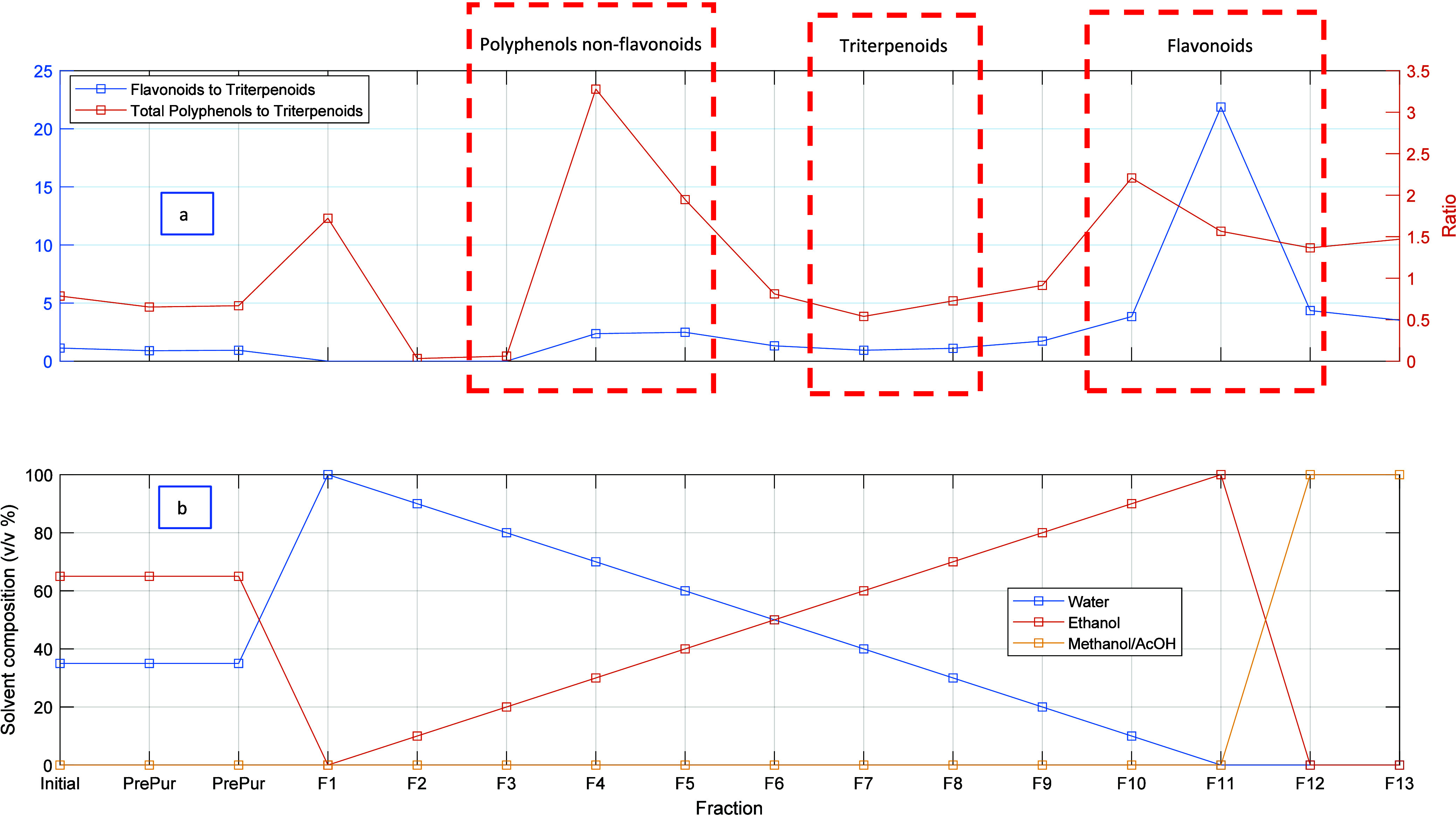
Illustration of a typical desorption step considered in the multicycle sorption/desorption fractionation of VR2 SS1 olive leaf extract using a pyridyl-functionalized adsorbent and hydroalcoholic solvents. The process involves a solvent gradient desorption at 45 °C starting from the column previously loaded with the crude extract or a preprocessed fraction (see Figure 5). A total of 13 different fractions were produced in this run. (a) Composition of each fraction concerning nonflavonoid polyphenols, triterpenoids, and flavonoid polyphenols and (b) solvent gradient used in this experiment.

Figure 10 illustrates the outcomes of the separation of polyphenols and triterpenoids in the VR2 SS1 olive leaf extract, employing a multistep sorption/desorption process in conjunction with the developed pyridyl-functionalized adsorbent. The experiment yielded 13 distinct fractions, whose composition with respect to nonflavonoid polyphenols (e.g., phenolic acids and secoiridoids), flavonoids (glycosylated flavonoids and the aglycone counterparts), and triterpene acids (e.g., maslinic and oleanolic acids) exhibited notable variation in response to the hydroalcoholic solvent utilized throughout the desorption process. It is noteworthy that the separation achieved for nonflavonoid polyphenols in the fractions desorbed with a low ethanol content (up to 40% ethanol in F1–F5) was particularly high. In contrast, the separation of flavonoids is optimized in the fraction desorbed with ethanol (F11), with a measured HPLC ratio of flavonoids to triterpenoids of approximately 23, as compared to the value of approximately 1 observed in the crude VR2 SS1 extract. In the range of 50% ethanol to 70% ethanol, triterpenoid desorption was observed, resulting in a reduction in the separation of polyphenols. These outcomes align with the simulations presented in Figure 4, which were based on the measured competitive adsorption isotherms.

**Figure 11:**
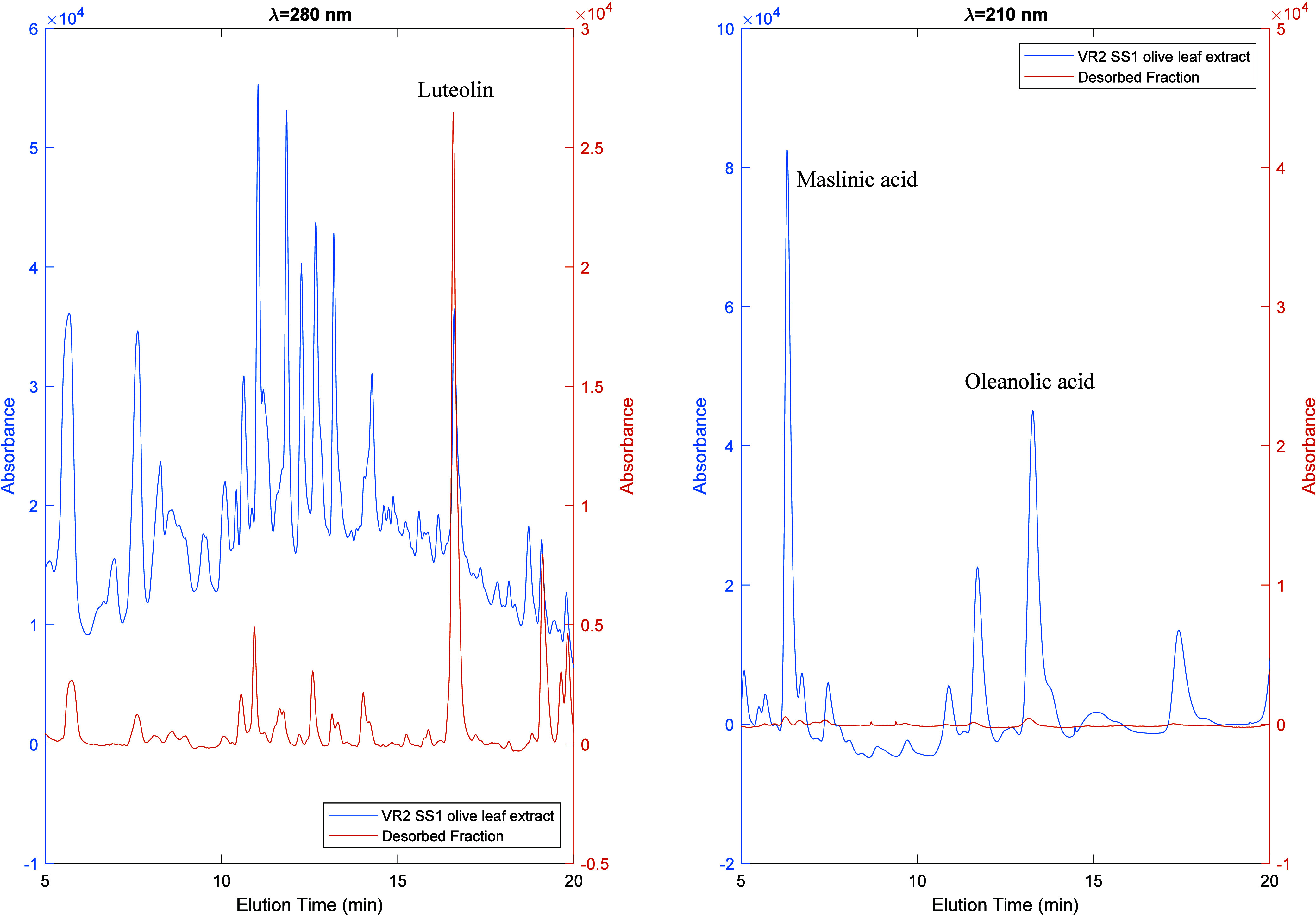
HPLC-DAD chromatograms for the crude VR2 SS1 olive leaf extract (blue line) and a fraction desorbed with ethanol (orange line) according to the solvent gradient method shown in Figure 10. The left plot corresponds to the analysis of the polyphenols in the samples (λ = 280 nm), while the triterpene composition (λ = 210 nm) is shown in the right plot. These results show the huge enrichment of luteolin and a significant decrease of triterpenes in the desorbed fraction as compared to the initial leaf extract.

Figure 11 provides compelling evidence to support the findings presented in Figure 10 regarding the separation of polyphenols and triterpenoids in the VR2 SS1 olive leaf extract. This is illustrated through a comparison of the HPLC analyses of a selected fraction (F11) and the crude extract. The left plot in Figure 11 shows the analysis for polyphenols (λ = 280 nm), while the triterpene composition (λ = 210 nm) is shown in the right plot. These results show the huge enrichment of luteolin and a significant decrease of triterpenes in the desorbed fraction compared to the initial leaf extract.

Table 1 provides an overview of various works that explore sorption/desorption systems related to the separation and purification of target compounds in olive tree byproducts. This comparison encompasses the type of adsorbent employed, the solvents utilized for sorption and desorption, and the objective of the process concerning the addressing of the olive tree subproducts. A number of systems employ commercial resins in the recovery of phenolic compounds from aqueous solutions, typically in the context of olive mill wastewater or olive brine storage.^26−30^ In other works, the objective is to quantify or extract contaminants or trace molecules in olive oil.^24,25^ Additionally, other research addresses the purification of target compounds in ethyl acetate extracts of olive leaf, as well as temperature-swing sorption/desorption processes utilizing ethyl acetate as the solvent.^22,23^ The present work deals with the separation of phenolic acids, phenylethanoids, secoiridoids, glycosylated and aglycone flavonoids, and triterpenoids in olive leaf industrial extracts. This is achieved through the utilization of ethanol/water mixtures at high alcohol contents (e.g., 80/20) and a solvent-gradient desorption process employing hydroalcoholic solvents. Indeed, the use of a pyridyl-functionalized adsorbent with hydroalcoholic solvents is adopted here due to the industrial practice for the production of olive leaf extracts (hydroalcoholic olive leaf extracts are particularly rich in a variety of phenolic compounds), as well as the greater simplicity of the fractionated product application in the feed, food, or cosmetic industries. Considering the final use of the fractions, the process can be redesigned to use less EtOH, depending on the separation and purification requirements. Increasing the global desorption temperature or using different temperature profiles along the various solvent gradient regions within the limits that prevent degradation of bioactive compounds is one way to reduce EtOH consumption. If permitted by product applications and process constraints, additional acidification of the hydroalcoholic mixtures is another option that has the potential to aid desorption and thus contribute to lower ethanol consumption.

**Table 1:** Different Sorption/Desorption Systems Related to the Separation and Purification of Target Compounds in Olive Tree Subproducts

adsorbent	adsor. solv.	desorp. solv.	observations	ref.
MIP-Oleuropein	ethyl acetate	ethyl acetate	temperature-swing process. Purification of oleuropein in olive leaf	(22)
MIP-Oleuropein	ethyl acetate	ethyl acetate	temperature-swing process. Studies with standard molecules	(23)
MIP-Luteolin				
MIP-Pinoresinol				
MIP-Itaconic acid	hexane	hexane dichloromethane, methanol	solid-phase extraction (SPE) for extraction of dimethoate from olive oil	(24)
MIP-3-(mercaptopropyl) trimethoxysilane	olive oil/Triton X-100 spiked with Cd (II)	aqueous solution of HCl 3 M	solid-phase extraction (SPE) adsorption of Cd(II) from vegetable oils	(25)
amberlite macroporous resins (XAD4, XAD16N, XAD7HP, and FPX66)	resins in storage brines	ethanol	adsorption of bitter and/or high-value phenolic compounds (oleuropein, oleacein, hydroxytyrosol, etc.) from whole olives during typical brine storage	(26)
resin AmberLite XAD16 N	aqueous solutions	water/ethanol 50/50	recovery of olive leaf phenols	(27,28)
resin XAD16	olive mill wastewater	acidified ethanol	recovery of olive phenols from olive mill wastewater	(29)
resins XAD4, XAD16, FPX 66	olive mill wastewater	ethanol/isopropanol 50/50 (v/v)	recovery of olive phenols from olive mill wastewater	(30)
pyridyl-functionalized adsorbent imprinted with quercetin	ethanol/water mixtures at high alcohol contents (e.g., 80/20)	hydroalcoholic gradient starting with water up to alcohol	separation of phenolic acids, phenylethanoids, secoiridoids, glycosylated and aglycone flavonoids, and triterpenoids in olive leaf industrial extracts	this work

The developed separation process is feasible when working with industrial olive leaf extracts at high concentrations (e.g., up to 10 mg/mL) due to the strong binding capacity of the pyridyl-functionalized adsorbent for many polyphenols, even when using aqueous mixtures with a large alcoholic content (e.g., loading solvents with an ethanol fraction higher than 50% v/v). DFT calculations indicate that the adsorption of flavonoids on pyridine nitrogen exhibits a lower energy than on other N-containing systems, such as pyrrole nitrogen and graphite nitrogen. This is thought to be due to the favorable charge accumulation on pyridine nitrogen compared to other N species, which makes it a highly favorable site for chemical adsorption of many flavonoids.

## 4 Conclusions

A tailor-made adsorbent functionalized with pyridyl moieties was used for the separation of a range of bioactive compounds present in olive leaves. This approach was developed with the goal of using only hydroalcoholic solvents and involved a combination of sorption and desorption processes.

The competitive binding isotherms of mixtures of vanillic acid, oleuropein, quercetin, maslinic acid, and oleanolic acid in water/ethanol solvents were measured and used to design sorption/desorption conditions that would facilitate the separation of the different kinds of bioactive compounds. Fractions comprising varying proportions of specific compounds were isolated from a polyphenol-rich industrial olive leaf extract. For example, oleuropein, with an approximate content of 80%, was obtained, corresponding to an enrichment factor of approximately four in comparison with the crude extract. Glycosylated flavonoids were separated at a content of around 60% (an enrichment factor of approximately 12), and aglycone flavonoids were separated at a concentration of approximately 83% (an enrichment factor of approximately 49). Conversely, the separation of polyphenols and triterpene acids was achieved by utilizing an olive leaf industrial extract with a high triterpene content as the starting material. The flavonoid/triterpenoid ratio in the isolated fractions was increased to approximately 23, which is a significant improvement over the ratio of approximately 1 observed in the crude extract. Furthermore, the separation of luteolin in the triterpene-rich extract was accomplished with an enrichment factor of approximately 7.

The pyridyl-based adsorbent and sorption/desorption approaches developed permit the separation of bioactive compounds in olive leaf extracts when operated at high concentrations and utilizing solely hydroalcoholic solvents, thereby facilitating more sustainable processing with diminished toxicological impact. The developed methodology exhibits minimal energy consumption and operates across a temperature range that prevents the degradation of the bioactive compounds. Furthermore, this enables the advancement of green practices and process intensification.

## Supporting Information Available

Molecular structures for representative bioactive compounds found in olive leaf (Figures S1–S6); overview for the working conditions considered in the analysis of the competitive sorption/desorption of polyphenols and triterpene acids in the pyridyl-functionalized adsorbent (Table S1); fitting results with the diverse isotherm models considered for the sorption of polyphenols and triterpene acids in the pyridyl-functionalized adsorbent (Tables S2–S6); estimated value for the quercetin adsorbed in the particles through a noncompetitive mechanism in relation to the other compounds (Table S7); and estimated change of isotherm parameters with the composition of the ethanol/water mixtures (Figure S7) (PDF)
