# Supplementary material for: Separation of Bioactive Compounds in Olive Leaf with a Pyridyl-Functionalized Adsorbent and Hydroalcoholic Solvents
Source: Ind Eng Chem Res. 2025 Feb 26;64(10):5575–88. doi: 10.1021/acs.iecr.4c04622 (PMC11907406; doi:10.1021/acs.iecr.4c04622)
Supplement: Supplementary file 1 — ie4c04622_si_001.pdf [file ie4c04622_si_001.pdf]

Supporting Information

Separation of Bioactive Compounds in Olive Leaf with a Pyridyl-Functionalized Adsorbent and Hydroalcoholic Solvents

Elchin Bilalov[1], Cláudia Martins[1], Mário Rui P. F. N. Costa[2], and Rolando C. S. Dias[1*]

[1]Centro de Investigação de Montanha (CIMO), Instituto Politécnico de Bragança, Campus de Santa Apolónia, 5300-253 Bragança, Portugal

[2]LSRE, Faculdade de Engenharia da Universidade do Porto, Rua Roberto Frias s/n, 4200-465 Porto, Portugal

* Correspondence to Rolando C. S. Dias, rdias@ipb.pt

This information is available free of charge via the Internet at http://pubs.acs.org/

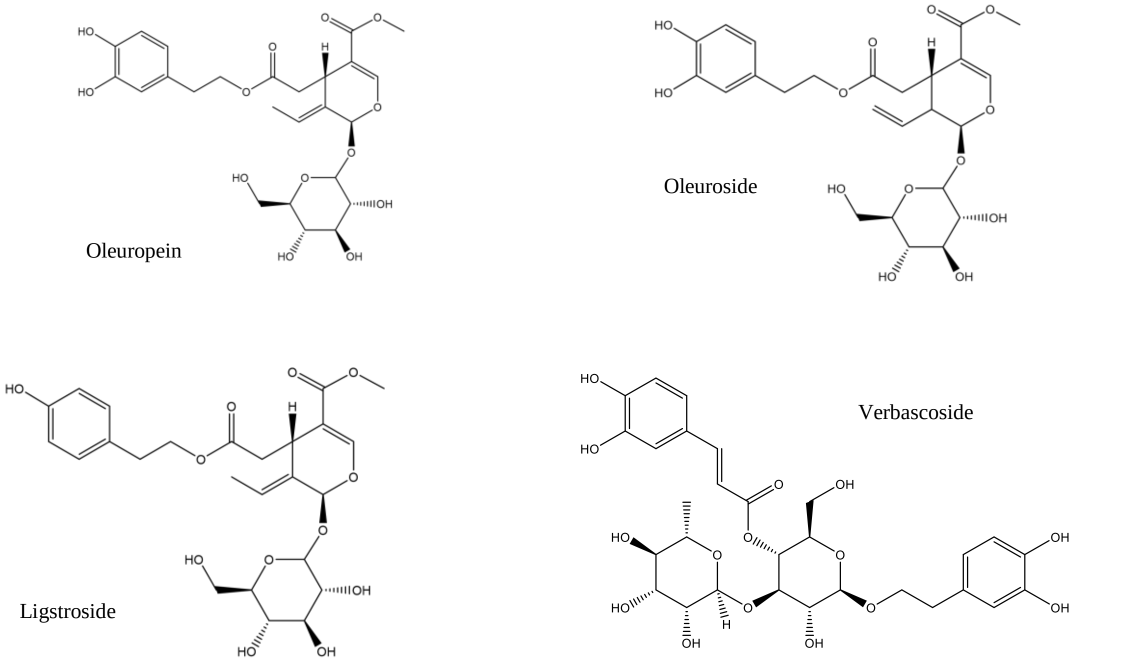

Figure S1: Molecular structures for representative bioactive compounds found in olive leaf. Secoiridoids: oleuropein, oleuroside, ligstroside. Phenylethanoid glycosides: verbascoside.

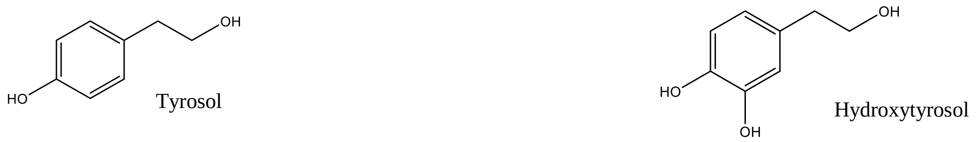

Figure S2: Molecular structures for representative bioactive compounds found in olive leaf. Phenylethanoids: tyrosol, hydroxityrosol.

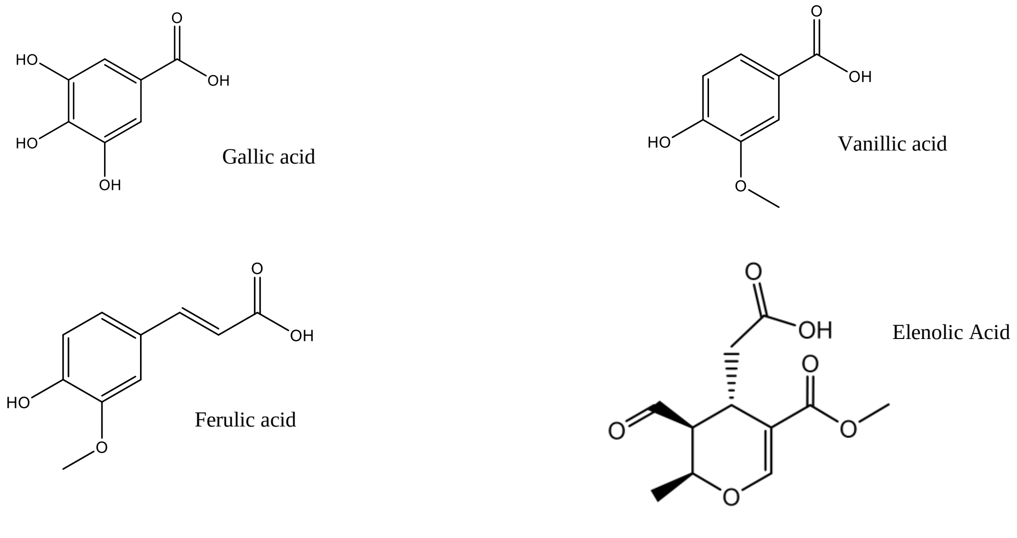

Figure S3: Molecular structures for representative bioactive compounds found in olive leaf. Phenolic acids: gallic acid, vanillic acid, ferulic acid. Non-phenolic acids: elenolic acid.

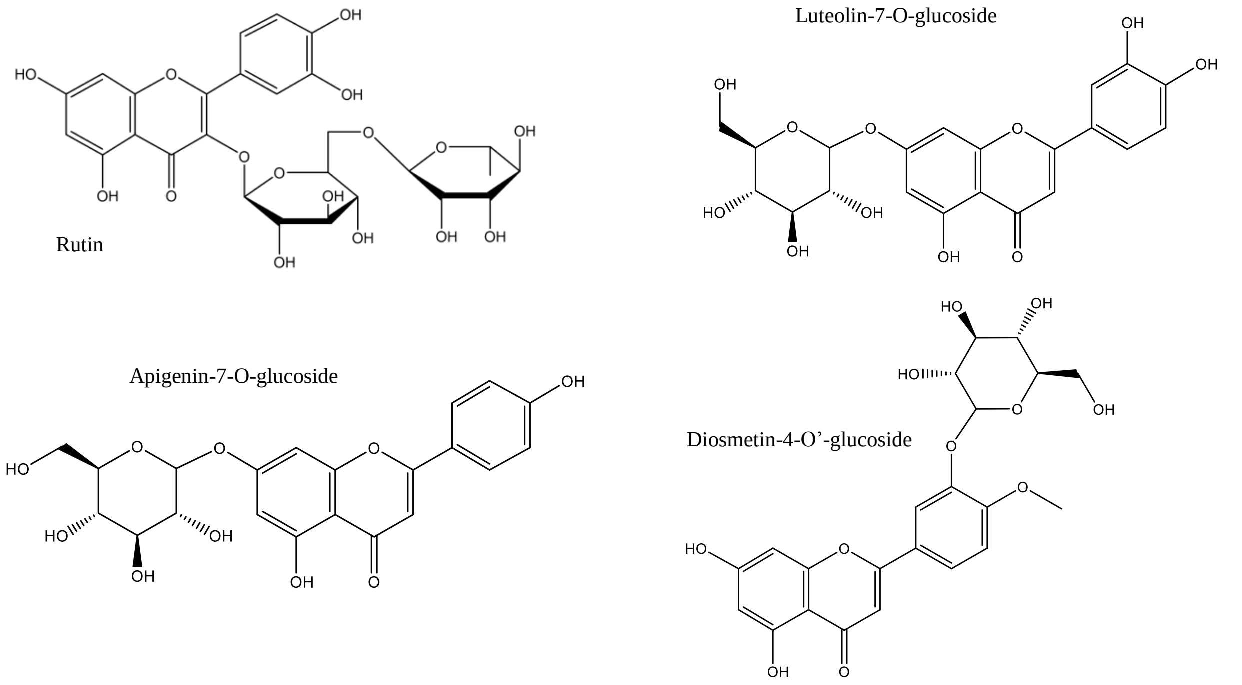

Figure S4: Molecular structures for representative bioactive compounds found in olive leaf. Glycosylated Flavonoids: rutin, luteolin-7-O-glucoside, apigenin-7-O-glucoside, diosmetin-4-O'-glucoside.

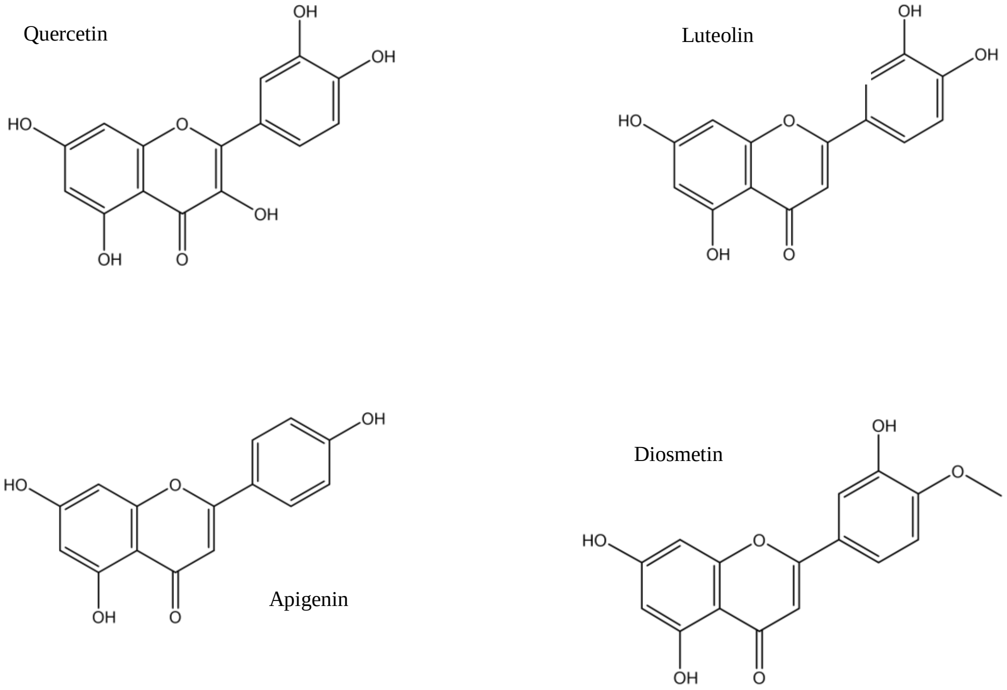

Figure S5: Molecular structures for representative bioactive compounds found in olive leaf. Aglycone Flavonoids: quercetin, luteolin, apigenin, diosmetin.

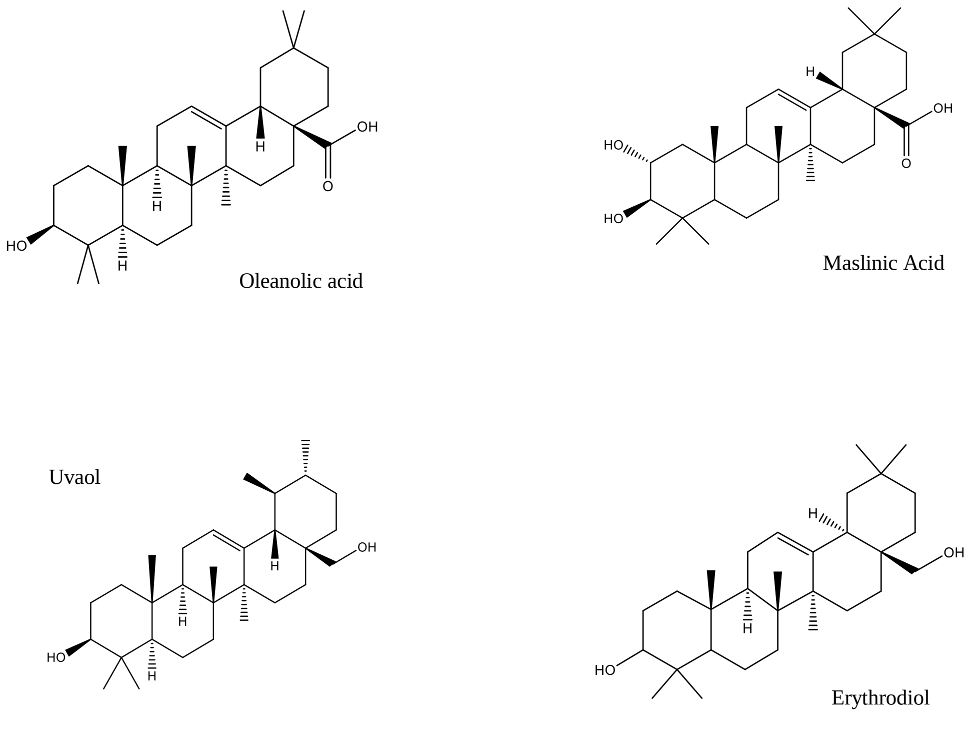

Figure S6: Molecular structures for representative bioactive compounds found in olive leaf. Triterpene acids: oleanolic acid, maslinic Acid. Triterpenoids: uvaol, erythrodiol.

Table S1: Overview for the working conditions considered in the analysis of the competitive sorption/desorption of polyphenols and triterpene acids. The solvent composition refers to volumetric amounts (v/v) and the concentration applies to each one of the five compounds considered. Sorption/desorption runs were performed at 25 ºC.

Solvent	Initial concentration range (mM)
EtOH/Water 100/0	0.75 to 3.0
EtOH/Water 80/20	0.1 to 2.0
EtOH/Water 65/35	0.125 to 0.5
EtOH/Water 50/50	0.03125 to 0.125

Table S2. Isotherm model parameters obtained for the fitting of the experimental data concerning the competitive adsorption of quercetin, vanillic acid, oleuropein, maslinic acid and oleanolic acid in the molecularly imprinted adsorbent. Results with the individual Langmuir model (Eq. (1) in the main text) are here presented for different working solvents. The function *lsqnonlin* of MATLAB was used with the fitting calculations.

		Quercetin	Vanillic Acid	Oleuropein	Maslinic Acid	Oleanolic Acid
EtOH/W 100/0	q_m	224.93	1902.49	235.53	3233.73	3613.74
	k_L	0.56	0.01	0.11	4.10E-03	3.70E-03
	$q_m \times k_L$	125.96	19.02	25.91	13.26	13.37
EtOH/W 80/20	q_m	206.65	37.09	16.51	1077.43	1673.05
	k_L	0.53	0.34	0.68	0.01	9.00E-03
	$q_m \times k_L$	109.52	12.61	11.23	10.77	15.06
EtOH/W 65/35	q_m	163.01	418.74	535.33	1849.94	1784.95
	k_L	1.74	0.05	0.03	0.01	0.01
	$q_m \times k_L$	283.64	20.94	16.06	18.50	17.85
EtOH/W 50/50	q_m	5.91	1.64	0.88	1.59	2.36
	k_L	905.51	49.73	53.16	48.73	72.52
	$q_m \times k_L$	5351.56	81.56	46.78	77.48	171.15

Table S3. Fitting results with the competitive Langmuir model (Eq. (3)). The function *lsqnonlin* of MATLAB was used with the fitting calculations.

Solvent	Parameter	Quercetin	Vanillic Acid	Oleuropein	Maslinic Acid	Oleanolic Acid
EtOH/W 100/0	q_m	1792.26	813.33	894.74	290.95	423.69
	k_L	0.06	0.03	0.04	0.06	0.05
	$q_m \times k_L$	107.54	24.40	35.79	17.46	21.18
EtOH/W 80/20	q_m	666.29	439.11	387.09	89.47	89.94
	k_L	0.16	0.03	0.02	0.12	0.15
	$q_m \times k_L$	106.61	13.17	7.74	10.74	13.49
EtOH/W 65/35	q_m	1186.58	643.63	520.27	290.66	110.19
	k_L	0.22	0.04	0.03	0.06	0.2
	$q_m \times k_L$	261.05	25.75	15.61	17.44	22.04
EtOH/W 50/50	q_m	661.84	438.04	386.41	59.53	1.75
	k_L	13.88	0.61	0.34	4.12	269.03
	$q_m \times k_L$	9186.34	267.20	131.38	245.26	470.80

Table S4. Fitting results with the extended competitive Langmuir model (Eqs. (5) - (6)). The function *fmincon* of MATLAB was used with the fitting calculations.

		Quercetin	Vanillic Acid	Oleuropein	Maslinic Acid	Oleanolic Acid
EtOH/W 100/0	q_m	5119.49	2024.78	2119	121.43	852.58
	k_L	0.02	0.01	0.02	0.16	0.02
	$q_m \times k_L$	102.39	20.25	42.38	19.43	17.05
EtOH/W 80/20	q_m	780.83	328.05	275.3	80.74	96.72
	k_L	0.14	0.04	0.03	0.13	0.14
	$q_m \times k_L$	109.32	13.12	8.26	10.50	13.54
EtOH/W 65/35	q_m	1694.85	656.01	373.6	578.68	83.69
	k_L	0.16	0.03	0.04	0.03	0.26
	$q_m \times k_L$	271.18	19.68	14.94	17.36	21.76
EtOH/W 50/50	q_m	38.78	15.84	2.01	2.18	18.75
	k_L	254.73	17.68	70.89	120.14	27.15
	$q_m \times k_L$	9878.43	280.05	142.49	261.91	509.06

Table S5. Fitting results with the individual Freundlich model (Eq. (2)). The function *lsqnonlin* of MATLAB was used with the fitting calculations.

		Quercetin	Vanillic Acid	Oleuropein	Maslinic Acid	Oleanolic Acid
EtOH/W 100/0	n	1.52	0.96	1.17	0.78	0.77
	K_F	77.73	18.20	23.99	10.46	10.65
EtOH/W 80/20	n	1.38	1.23	1.31	1.32	1.18
	K_F	67.26	8.95	6.07	6.09	7.33
EtOH/W 65/35	n	1.17	1.02	1.00	0.73	0.78
	K_F	169.20	19.22	13.32	22.78	25.06
EtOH/W 50/50	n	3.02	1.76	1.98	1.84	1.98
	K_F	25.47	6.78	2.96	5.95	8.73

Table S6. Fitting results with the individual competitive Freundlich model (Eq. (4)). The function *fmincon* of MATLAB was used with the fitting calculations.

		Quercetin	Vanillic Acid	Oleuropein	Maslinic Acid	Oleanolic Acid
EtOH/W 100/0	n	1.53	1.04	1.23	0.72	0.71
	K_F	82.71	23.68	33.15	7.16	7.13
EtOH/W 80/20	n	1.69	1.55	1.82	0.98	0.75
	K_F	75.90	22.09	19.54	8.61	6.02
EtOH/W 65/35	n	1.22	1.03	0.96	0.58	0.63
	K_F	148.69	20.43	13.91	12.05	17.80
EtOH/W 50/50	n	3.46	2.62	2.73	2.62	2.88
	K_F	50.17	16.32	11.96	15.72	16.83

Table S7. Estimated value for the quercetin adsorbed in the MIP particles through a non-competitive mechanism in relation to the other compounds. This particular non-competitive adsorption of quercetin is conceived as a possible consequence of the molecular imprinting effect. These estimated were obtained through the fitting results with the extended competitive Langmuir model (Eqs. (5) - (6)).

Quercertin	EtOH/W 100/0	EtOH/W 80/20	EtOH/W 65/35	EtOH/W 50/50
Non-competitive adsorption (μmol/g)	1.68e+00	3.19e-04	2.93e+00	6.24e-04
Non-competitive adsorption (%)	1.64e-04	2.04e-07	8.66e-04	8.04e-06

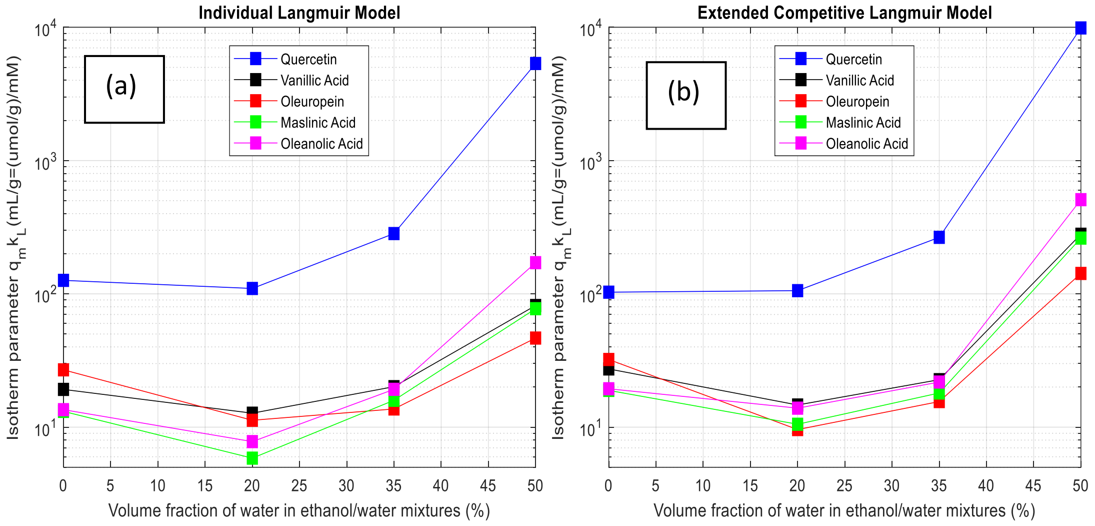

Figure S7: Experimentally estimated values for the parameter $q_m \times k_L$ (μmol/g $\times$ mM^{-1}) with four different compositions of the solvent EtOH/Water (100/0, 80/20, 65/35 and 50/50 v/v) for the competitive adsorption of vanillic acid, oleuropein, quercetin, maslinic acid and oleanolic acid in the pyridyl functionalized adsorbent particles. Estimations using the fitting of the experimental data do the individual Langmuir model are presented in (a), while the extended competitive Langmuir model was used to obtain the estimated in (b).
